# Floral Guide

**Published:** 1869-01

**Authors:** 


### FLORAL GUIDE.

We have just received *Vick's Floral Guide* for 1869. This is a beautifully printed and illustrated catalogue of seeds, and guide in the flower garden. It is the most complete work of the kind we have ever seen; it embraces about one hundred pages, beautifully illustrated, with over one hundred and fifty fine wood engravings of flowers, plants and vegetables, and an elegant colored plate, a boquet of flowers. It contains accurate descriptions of the leading floral treasures of the world, with plain and full directions for sowing seed, transplanting and after culture. The author says:

"The *Floral Guide* is published for the benefit of my customers, to whom it is sent free without application, but will be forwarded to all who apply by mail for ten cents, which is not half the cost. Address, JAMES VICK, Rochester, N. Y."

